# Antibiotic prophylaxis for surgical site infections as a risk factor for infection with *Clostridium difficile*

**DOI:** 10.1371/journal.pone.0179117

**Published:** 2017-06-16

**Authors:** Aubrey Balch, Aaron M. Wendelboe, Sara K. Vesely, Dale W. Bratzler

**Affiliations:** University of Oklahoma Health Sciences Center, College of Public Health, Oklahoma City, United States of America; University of Calgary, CANADA

## Abstract

**Objective:**

We aimed to measure the association between 2013 guideline concordant prophylactic antibiotic use prior to surgery and infection with *Clostridium difficile*.

**Design:**

We conducted a retrospective case-control study by selecting patients who underwent a surgical procedure between January 1, 2012 and December 31, 2013.

**Setting:**

Large urban community hospital.

**Patients:**

Cases and controls were patients age 18+ years who underwent an eligible surgery (i.e., colorectal, neurosurgery, vascular/cardiac/thoracic, hysterectomy, abdominal/pelvic and orthopedic surgical procedures) within six months prior to infection diagnosis. Cases were diagnosed with *C*. *difficile* infection while controls were not.

**Methods:**

The primary exposure was receiving (vs. not receiving) the recommended prophylactic antibiotic regimen, based on type and duration. Potential confounders included age, sex, length of hospital stay, comorbidities, type of surgery, and prior antibiotic use. Crude and adjusted odds ratios (OR) and 95% confidence intervals (CI) were calculated using logistic regression.

**Results:**

We enrolled 68 cases and 220 controls. The adjusted OR among surgical patients between developing *C*. *difficile* infection and not receiving the recommended prophylactic antibiotic regimen (usually receiving antimicrobial prophylaxis for more than 24 hours) was 6.7 (95% CI: 2.9–15.5). Independent risk factors for developing *C*. *difficile* infection included having severe comorbidities, receiving antibiotics within the previous 6 months, and undergoing orthopedic surgery.

**Conclusions:**

Adherence to the recommended prophylactic antibiotics among surgical patients likely reduces the probability of being case of *C*. *difficile*. Antibiotic stewardship should be a priority in strategies to decrease the morbidity, mortality, and costs associated with *C*. *difficile* infection.

## Introduction

Surgical site infections are the leading cause of hospital-acquired infections[1] and are associated with excess hospital costs [2]. In 2002, the Surgical Infection Prevention Project, and the subsequent Surgical Care Improvement Project were implemented to decrease the morbidity and mortality associated with surgical site infections following a surgical procedure [3–5]. The project focused on the appropriate selection, timing of administration and dosage of the antibiotic while balancing the risks such as allergic reactions and *Clostridium difficile* infections [2, 6].

Since 2000, a new hypervirulent strain of *C*. *difficile* (variously described as BI, NAP1, or ribotype 027) has significantly increased the incidence and mortality rates of antibiotic-associated diarrhea and pseudomembranous colitis, especially among hospitalized patients [2]. Southern et al. reported that patients who underwent surgical procedures were also at risk (0.2%–8%) for developing *C*. *difficile* infection [7]. Reported risk factors for postoperative *C*. *difficile* infection include age, antibiotic use within 30 days prior to operation, high-risk antibiotic use within 30 days prior to operation, proton-pump inhibitor use within 10 days prior to operation, prior hospitalization, decreased immunity, and low serum albumin level on admission [7–9].

A few studies have reported a decreased incidence of *C*. *difficile* infection among surgical patients for whom a protocol was followed which reduced peri-operative antibiotic use [10–16]. Among the studies reporting decreased risk of *C*. *difficile* infection, the magnitude of reduction ranged from two to six fold.[10–13] Three of these studies were designed to demonstrate the effectiveness of new antibiotic policies recommending narrower-spectrum antibiotics and lower doses [11–13], the most restrictive of which allowed for a single-dose of gentamicin and amoxicillin antibiotic prophylaxis regimen for hip hemiarthroplasty in surgical patients [13].

Antibiotics associated with a higher potential to induce *C*. *difficile* infection include aminopencillins, cephalosporins, clindamycin, and fluoroquinolones [10]. Prolonged courses of antibiotics or the use of two or more antibiotics in combination increases the risk of *C*. *difficile* infection [11]. The general recommendation that pertains to our study is that surgical patients be given the recommended type of antibiotic based on the type of surgical procedure and that surgical patients be given 24 hours or less of antibiotic prophylaxis after the end of surgery [2, 17]. These national recommendations were detailed in a guideline published by the American Society of Health-System Pharmacists (ASHP) Report in 2013 [17]. For each surgical procedure, specific antibiotics are recommended and additional recommendations are provided for patients with β–Lactam allergies. The guideline also states that a single-dose of prophylactic antibiotics is usually sufficient, but the duration of prophylactic antibiotics for all procedures should not exceed 24 hours [17]. The World Health Organization (WHO) and the American College of Surgeons also released similar recommendations regarding the optimal timing and dose of surgical antibiotic prophylaxis [18–20]. The WHO’s recommendations were stratified by preoperative (recommendations 9 & 10) and intraoperative/postoperative (recommendation 16) measures for surgical site infection prevention [19, 20].

We conducted a case-control study aimed at measuring the association between the adherence to these updated recommendations regarding peri-operative antibiotic use and developing *C*. *difficile* infection. We hypothesized that patients with *C*. *difficile* infection would more frequently have received antibiotics longer than the recommended 24-hour duration compared to patients who did not have *C*. *difficile* infection.

## Materials and methods

### Study design

We conducted a case-control study of surgical patients at a large urban community hospital by selecting eligible surgical patients between January 1, 2012 and December 31, 2013. The timeframe was chosen as the most recent period in which there were at least 100 cases. Eligible surgeries included colorectal, neurosurgery, vascular/cardiac/thoracic, hysterectomy, abdominal/pelvic and orthopedic surgical procedures and were identified by ICD-9 procedure code as listed in S5 Table. Cases were defined as patients 18 years of age or older diagnosed with *C*. *difficile* infection who underwent an eligible surgery within six months prior to infection diagnosis. *C*. *difficile* infection was defined by ICD-9 code 008.45 as found in the medical record. Controls were defined as patients 18 years of age or older who underwent an eligible surgery but did not get infected with *C*. *difficile*. Three controls for each case were selected.

Initially, we collected data on 100 cases and 300 controls. During the analysis phases, we applied a restriction criterion that the surgery must have occurred either on the day of or day following admission. (Our rationale for imposing this restriction criterion is that increased length of stay can be both a risk factor for *C*. *difficile* infection and also a consequence of being infected with *C*. *difficile* [15].) Thus 68 (68.0%) cases and 220 (73.3%) controls were included the primary analyses. The results comparing the original 400 participants and the subset of 288 participants restricted by day of surgery are presented in the Appendix (S1–S4 Tables). This study was approved by the University of Oklahoma Health Sciences Institutional Review Board (IRB# 3859). The IRB waived the need for consent to access the patients’ medical records. Initially, we had access to patient identifying information, but we deidentified the data after they were cleaned and we began the analysis process.

### Data collection

Prior to conducting the study, we consulted CDC Division of Healthcare Quality Promotion (DHQP) seeking guidance on all aspects of data collection, including recommended variables, their classification and categorization. DHQP sent us data collection instruments they had used in previous studies, which we tailored to our current study. The primary exposure was a composite binary variable of whether the ASHP recommended [17] antibiotic regimen was given to surgical patients for their operation. Type and duration of antibiotic administration were collected to determine if recommendations were followed. After data collection was complete, we went through each case and control individually to determine if the patient received the recommended prophylactic antibiotic based on two criteria: 1) the patient must have received the recommended antibiotic based on the surgical procedure and 2), all antibiotics must have been stopped with 24 hours of surgery (S5 Table). Additional information collected included date of birth, sex, length of hospital stay (calculated using admission and discharge date), primary diagnosis, comorbidities, type of surgical procedure, date of surgical procedure, hospital room number, and, when available, a list of antibiotics received within six months before hospitalization.

Based on input from CDC/DHQP (oral communication) and to control for patients’ potential predisposition to get *C*. *difficile* enterocolitis, comorbidities associated with *C*. *difficile* infection were classified into three mutually exclusive severity levels. Level 1 included myocardial infarction, chronic lung disease, chronic liver disease, peripheral vascular disease, cerebrovascular disease, and congestive heart failure. Level 2 included diabetes with end organ damage, acute or chronic renal disease, lymphoma, cancer, metastatic solid tumor, and tumor without metastasis. Patients with more than one of the above comorbidities (regardless of the severity level) were classified into a higher severity category, severity level 3.

### Statistical methods

Summary statistics were calculated to describe the data. Odds ratios (OR) and 95% confidence intervals (CI) were calculated for categorical variables and student’s t-test was used for continuous variables. Confounding was assessed for the following covariates: age, sex, length of stay, having a comorbidity, having a severe comorbidity, and whether antibiotics had been given six months prior to the surgery. Unadjusted ORs and 95% CIs were calculated between each variable and case status. An adjusted logistic regression model was built using forward variable selection. Adjusted ORs and 95% CIs were calculated using logistic regression. Hosmer and Lemeshow goodness-of-fit statistics were used to build a best-fitting model. All first order interaction combinations among the variables in the main effects models were assessed. An alpha = 0.05 was established for determining significance of main effects and interaction terms. Confounding was evaluated and considered present if a risk factor changed the OR by 20%. The fit of the final model was assessed by the global null hypothesis. All analyses were conducted in SAS 9.2 (Cary, NC).

## Results

Among the 68 cases and 220 controls who had their surgery either on the day of admission or the day following admission, 37 (54.4%) cases and 53 (24.1%) controls had one or more comorbidity. The distribution of comorbidities categorized into severity levels 1 and 2 is shown in Table 1.

**Table 1:** Distribution of comorbidities by case and control status among the full sample and patients whose surgery was on day 0 or day 1 of admission.

Comorbidities*		Cases		Controls	
		n	%	n	%
Severity Level 1					
	Myocardial infarction	2	2.9	5	2.3
	Chronic lung disease	10	14.7	12	5.5
	Chronic liver disease	1	1.5	3	1.4
	Peripheral vascular disease	5	7.4	3	1.4
	Congestive heart failure	8	11.8	10	4.5
	Cerebrovascular disease	5	7.4	7	3.2
Severity Level 2					
	Diabetes with end organ damage	9	13.2	1	0.5
	Acute to chronic renal disease	10	14.7	10	4.5
	Cancer**	9	13.2	13	5.9
None		31	45.6	167	75.9

*Comorbidities are not mutually exclusive. A patient may have more than one comorbidity among both severity levels 1 and 2.

**Cancer includes any form of cancer (e.g., tumor with or without metastasis or lymphoma)

The distributions of demographic and clinical risk factors stratified by case status are shown in Table 2 (continuous variables) and Table 3 (categorical variables). In the crude analyses, we found the odds of not receiving the recommended antibiotic prophylaxis regimen was six times greater among cases of *C*. *difficile* as compared to controls (OR = 6.0, 95% CI: 3.3, 10.8). Reasons for not receiving the recommended antibiotic regimen are as follows: received antibiotics longer than recommended (51.2%), received an antibiotic not within the recommended guidelines (11.6%), received no antibiotics (11.6%), received antibiotics shorter than recommended (4.7%), and reason not recorded (20.9%). Mean age (p = 0.49) and the mean number of prophylactic drugs for the present surgery (p = 0.18) did not differ between cases and controls. However, the mean length of stay, the mean number of prior antibiotics prescribed, and the mean days on prior antibiotics were significantly greater for cases than controls (p<0.01 for each variable). Sex and surgery type were not associated with case status while being prescribed antibiotics in the six months prior to surgery (OR = 15.1, 95% CI: 7.6, 29.8) was. In addition, there may be a dose response relationship in the severity of having a comorbidity with the odds of having *C*. *difficile* infection as the OR for having a severity level 1 comorbidity was 2.0 (95% CI: 0.92, 4.5), a severity level 2 comorbidity was 2.7 (95% CI: 1.0, 7.2) and a severity level three comorbidity was 10.2 (95% CI: 4.3, 24.1).

**Table 2:** Comparison of means for age, length of hospital stay, number of previous prophylactic antibiotics, and the number of days on prophylactic antibiotics by case status.

Variables	Case		Control		P-value**		
	Mean	Median	Mean	Median		OR	95% CI
Age (years)	65.9	70	64.5	66.5	0.49	1.0	0.99, 1.0
Length of stay (days)	18.0	11	5.7	2	<0.01	1.0	1.0, 1.1
Number of prophylactic antibiotics for current surgery	1.4	1	1.2	1	0.18	1.4	0.94, 2.1
Number of prior antibiotics*	1.4	0	0.2	0	<0.01	2.0	1.5, 2.6
Days on prior antibiotics	7.4	0	0.8	0	<0.01	1.1	1.1, 1.2

Abx: antibiotics

*Number of antibiotics taken up to six months prior to surgery

**p-value comparing means

**Table 3:** Unadjusted odds ratios (OR) and 95% confidence intervals (CI) for demographic and clinical risk factors.

Risk Factor (Categorical)		Cases			Controls			OR	95% CI
		n	%		n	%			
Received recommended antibiotic prophylaxis									
	No	43	63.2		49	22.3		6.0	3.3, 10.8
	Yes	25	36.8		171	77.7		Ref	…
Sex									
	Male	36	52.9		97	44.1		1.4	0.82, 2.5
	Female	32	47.1		123	55.9		Ref	…
Antibiotics given 6 months prior									
	Yes	39	57.4		18	8.2		15.1	7.6, 29.8
	No	29	42.7		202	91.8		Ref	…
Comorbidities									
	Severity level 1	11	16.2		29	13.2		2.0	0.92, 4.5
	Severity level 2	7	10.3		14	6.4		2.7	1.0, 7.2
	Severity level 3	19	27.9		10	4.6		10.2	4.3, 24.1
	None	31	45.6		167	75.9		Ref	…
Surgery									
	Colorectal	8	11.8		24	10.9		0.96	0.38, 2.4
	Orthopedic	15	22.1		43	19.6		1.0	0.48, 2.1
	Vascular, cardiac, thoracic	16	23.5		56	25.5		0.82	0.40, 1.7
	Neurosurgery	4	5.9		10	4.6		1.2	0.33, 4.0
	Hysterectomy	0	0.0		15	6.8		Und	…
	Abdominal, pelvic	25	36.8		72	32.7		Ref	…

Ref: reference group; Und: undefined.

The results from the multivariate analysis are shown in Table 4. The OR for not receiving the recommend antibiotic regimen was 6.7 (95% CI: 2.9, 15.5). Other significant independent factors included having severity level 2 and level 3 comorbidities (OR = 3.8 and OR = 4.0, respectively), receiving antibiotics in the 6 months prior to surgery (OR = 19.1) and having an orthopedic surgery (OR = 3.4). In contrast, age and sex were not associated with case status. The control-subjects who underwent hysterectomy (n = 15) were excluded because of the lack of case-subjects having had a hysterectomy.

**Table 4:** Adjusted odds ratios (OR) and 95% confidence intervals (CI) for risk factors independently associated with case status.

Parameter		OR	95% CI	P-value
Non-recommended Antibiotic Use		6.7	2.9, 15.5	<0.01
Comorbidity Severity				
	Level 1	0.88	0.30, 2.6	0.82
	Level 2	3.8	1.1, 13.9	0.04
	Level 3	4.0	1.4, 11.7	0.01
Antibiotics given 6 months prior		19.1	8.0, 45.7	<0.01
Surgery Type				
	Colorectal	1.4	0.4, 4.7	0.55
	Orthopedic	3.4	1.1, 10.0	0.03
	Vascular, cardiac, thoracic	1.1	0.44, 2.9	0.81
	Neurosurgery	1.2	0.2, 6.4	0.87
	Abdominal, pelvic	Ref	…	…

Ref: reference group.

The results for each of the above measures were similar among the full sample when not restricted by hospital day that the surgery was performed. Corresponding tables are found in the supporting information (S1 Table, S2 Table, S3 Table and S4 Table). The adjusted OR for not receiving the recommended antibiotic prophylactic regimen among the full sample was 7.6, 95% CI: 3.9, 14.7.

## Discussion

Our findings strongly support the use of guideline concordant antibiotic prophylaxis to reduce the risk of *C*. *difficile* infection. Specifically, the odds of not receiving guideline concordant antibiotic prophylaxis were 6.7 times higher (95% CI: 2.9, 15.5) among those patients who developed an infection with *C*. *difficile* compared to those who did not. These results are robust to changes in model specifications and underlying assumptions about confounding from covariates. For example, when all subjects were included in the analyses, regardless of when their surgery occurred in relation to their admission date (affecting length of stay), the odds of not receiving recommended prophylaxis increased to 7.6 (95% CI: 3.9, 14.7) among cases of *C*. *difficile* as compared to controls. (S1–S4 Tables.) These findings are consistent with previous studies, such as O’Connor et al.’s report that the risk of developing *C*. *difficile* infections was significantly higher when patients were given antibiotics based on an old antibiotic policy which allowed the routine use of broad spectrum cephalosporins (RR = 3.24, 95% CI: 1.07, 9.84; p = 0.03) [11]. Similar associations between the lack of appropriate antibiotic prophylaxis and *C*. *difficile* infection (OR = 3.34, 95% IC: 1.66, 6.73) was reported among a population of pediatric surgical cases [14].

Since the early 1990s, studies have linked the use of surgical prophylactic antimicrobials and infection with *C*. *difficile*. Yee and colleagues demonstrated that postoperative *C*. *difficile* diarrhea occurred just as commonly in patients receiving prophylactic antibiotics as those patients receiving therapeutic antimicrobials [21]. In a small study, Mukhtar and colleagues found that 60% of postoperative patients who developed *C*. *difficile* infection had received prophylactic antimicrobials only, and of those 65% received more than three postoperative doses [22]. In patients undergoing abdominal hysterectomy, hip arthroplasty, craniotomy, or colon, cardiac, or vascular surgery, the incidence of *C*. *difficile* infection was just as common in patients receiving prophylactic antimicrobials as it was in patients receiving therapeutic antimicrobials [2]. The use of additional antimicrobials [23], broad spectrum antimicrobials, and prolonged prophylaxis [24] have all been associated with increased risk of *C*. *difficile* disease.

Prior to this study, it was evident efforts needed to be made to control the *C*. *difficile* infection rate among hospitalized patients. Carignan et al. reported a 21-fold increase in *C*. *difficile* infection (from 0.7/1000 to 14.9/1000) during the study period (1999–2005) [2]. Our findings contribute to the evidence that *C*. *difficile* infection is significantly associated with inappropriate prophylactic antibiotic use. The fact that virtually every surgical patient who receives antibiotics is at risk for *C*. *difficile* infection should be considered when selecting the patient-specific antibiotic regimen before surgery.

While our results help measure the impact of following recommendations for antibiotic prophylaxis among these surgical patients on the development of *C*. *difficile* infection, they do not attempt to evaluate the clinical reasons why the recommendations were not followed. However, we did strive to control for factors which may have made the case and control populations different. These factors include the severity of comorbid conditions and length of hospital stay, which affects a patient’s predisposition to *C*. *difficile* infection.

Previous studies have shown a number of surgical procedures to be associated with the development of *C*. *difficile* among those not receiving the recommended antibiotic prophylaxis regimen. Specifically, radical nephrectomy and radical cystectomy among urological cancer patients [16], colectomy, small-bowel resection, and gastric resection [15], and hypospadias/epispadias repair, removal of femur hardware, and repair of tendon contracture [14] have been highlighted. In our study, orthopedic surgical procedures had the strongest association with infection with *C*. *difficile* (OR = 3.4, 95% CI: 1.1, 10.0) among those not receiving the recommended antibiotic prophylaxis regimen.

The primary limitation to this case-control study relates to using medical records for data collection. We are limited by the amount of information entered into the electronic medical records and thus there is no meaningful distinction between a “no/absent” or “unknown” value for covariates. This impacted antibiotic exposure assessment by 1) uncertainty that every antibiotic and dose were entered into the record and 2) sometimes conflicting information regarding antibiotic administration prior to surgery when entered in multiple areas within the record. The quality of antibiotic exposure was more problematic for those used up to 6 months prior to surgery than for the current surgery. The antibiotics recorded were antibiotics only given at the health system at which the study was conducted. Antibiotics given at other facilities (both inpatient and outpatient) were most likely missed. However, these missing data are unlikely associated with case status and thus would probably bias the results towards the null. Another limitation stems from this study being conducted at a single hospital and thus the results may not be generalizable to other facilities.

The main results from our study supported our hypothesis that patients with *C*. *difficile* infection more frequently received antibiotics longer than the recommended 24-hour duration compared to patients who did not have *C*. *difficile* infection. These results were consistent across both crude and adjusted results as well as among patients whose surgery occurred within the first two days of hospital admission or during any time of hospital admission. These results may be helpful in supporting antibiotic stewardship strategies aimed at reducing the costs, morbidity, and mortality associated with *C*. *difficile* infection in surgical patients.

## Supporting information

S1 Table Distribution of comorbidities by case and control status among the full sample and patients whose surgery was on day 0 or day 1 of admission. (DOCX)

S2 Table Comparison of means for age, length of hospital stay, number of previous prophylactic drugs, and the number of days on prophylactic drugs by cases status. (DOCX)

S3 Table Unadjusted odds ratios and 95% confidence intervals (CI) for demographic and clinical risk factors. (DOCX)

S4 Table Adjusted odds ratios (OR) and 95% confidence intervals (CI) for risk factors independently associated with case status. (DOCX)

S5 Table List of eligible surgeries as defined by ICD-9 procedure code. (DOCX)
