# Supplementary material for: Antibiotic prophylaxis for surgical site infections as a risk factor for infection with Clostridium difficile
Source: PLoS One. 2017 Jun 16;12(6):e0179117. doi: 10.1371/journal.pone.0179117 (PMC5473553; doi:10.1371/journal.pone.0179117)
Supplement: S1 Table — (DOCX) [file pone.0179117.s001.docx]

**S1 Table. Distribution of comorbidities by case and control status among the full sample and patients whose surgery was on day 0 or day 1 of admission.**

| **Comorbidities*** | | | **Full Sample** | | | | **Subset Surgery upon Admission** | | | |
| --- | --- | --- | --- | --- | --- | --- | --- | --- | --- | --- |
|  |  |  | **Cases** | | **Controls** | | **Cases** | | **Controls** | |
|  |  |  | **n** | **%** | **n** | **%** | **n** | **%** | **n** | **%** |
|  | Severity Level 1 | |  |  |  |  |  |  |  |  |
|  |  | Myocardial infarction | 7 | 7.0 | 7 | 2.3 | 2 | 2.9 | 5 | 2.3 |
|  |  | Chronic lung disease | 15 | 15.0 | 16 | 5.3 | 10 | 14.7 | 12 | 5.5 |
|  |  | Chronic liver disease | 2 | 2.0 | 5 | 1.7 | 1 | 1.5 | 3 | 1.4 |
|  |  | Peripheral vascular disease | 7 | 7.0 | 4 | 1.3 | 5 | 7.4 | 3 | 1.4 |
|  |  | Congestive heart failure | 16 | 16.0 | 17 | 5.7 | 8 | 11.8 | 10 | 4.5 |
|  |  | Cerebrovascular disease | 8 | 8.0 | 9 | 3.0 | 5 | 7.4 | 7 | 3.2 |
|  | Severity Level 2 | |  |  |  |  |  |  |  |  |
|  |  | Diabetes with end organ damage | 9 | 9.0 | 2 | 0.7 | 9 | 13.2 | 1 | 0.5 |
|  |  | Acute to chronic renal disease | 10 | 10.0 | 12 | 4.0 | 10 | 14.7 | 10 | 4.5 |
|  |  | Cancer** | 12 | 12.0 | 21 | 7.0 | 9 | 13.2 | 13 | 5.9 |
|  | None | | 43 | 43.0 | 223 | 74.3 | 31 | 45.6 | 167 | 75.9 |

*Comorbidities are not mutually exclusive. A patient may have more than one comorbidity among both the

severity levels 1 and 2.

**Cancer includes any form of cancer (e.g., tumor with or without metastasis or lymphoma)
