# Supplementary material for: Antibiotic prophylaxis for surgical site infections as a risk factor for infection with Clostridium difficile
Source: PLoS One. 2017 Jun 16;12(6):e0179117. doi: 10.1371/journal.pone.0179117 (PMC5473553; doi:10.1371/journal.pone.0179117)
Supplement: S2 Table — (DOCX) [file pone.0179117.s002.docx]

**S2 Table. Comparison of means for age, length of hospital stay, number of previous prophylactic drugs, and the number of days on prophylactic drugs by cases status.**

| **Variables** | **Full Sample** | | | **Subset Surgery Upon Admission** | | |
| --- | --- | --- | --- | --- | --- | --- |
|  | **Case** | **Control** | **P-value** | **Case** | **Control** | **P –value** |
|  | Mean | Mean |  | Mean | Mean |  |
| Age (years) | 67.1 | 65.7 | 0.23 | 65.9 | 64.5 | 0.49 |
| Length of stay (days) | 20.5 | 7.0 | <0.01 | 18.0 | 5.7 | <0.01 |
| Number of prophylaxis drugs | 1.3 | 1.2 | 0.29 | 1.4 | 1.2 | 0.18 |
| Number of prior antibiotics | 1.3 | 0.2 | <0.01 | 1.4 | 0.2 | <0.01 |
| Days on prior antibiotics | 7.1 | 0.6 | <0.01 | 7.4 | 0.8 | <0.01 |
