# Supplementary material for: Antibiotic prophylaxis for surgical site infections as a risk factor for infection with Clostridium difficile
Source: PLoS One. 2017 Jun 16;12(6):e0179117. doi: 10.1371/journal.pone.0179117 (PMC5473553; doi:10.1371/journal.pone.0179117)
Supplement: S3 Table — (DOCX) [file pone.0179117.s003.docx]

**S3 Table. Unadjusted odds ratios and 95% confidence intervals (CI) for demographic and clinical risk factors.**

|  | | **Full Sample** | | | | | | **Subset based on Surgery upon Admission** | | | | | | | |
| --- | --- | --- | --- | --- | --- | --- | --- | --- | --- | --- | --- | --- | --- | --- | --- |
| **Risk Factor (Categorical)** | | **Cases** | | **Controls** | | **OR** | **95%CI** | **Cases** | | | **Controls** | | | **OR** | **95%CI** |
|  |  | **n** | **%** | **n** | **%** |  |  | **n** | **%** | | **n** | **%** | |  |  |
| Received recommended antibiotic prophylaxis | |  |  |  |  |  |  |  |  | |  |  | |  |  |
|  | No | 69 | 69.0 | 76 | 25.3 | 6.6 | 4.0, 10.8 | 43 | 63.2 | | 49 | 22.3 | | 6.0 | 3.3, 10.8 |
|  | Yes | 31 | 31.0 | 224 | 76.7 | Ref | … | 25 | 36.8 | | 171 | 77.7 | | Ref | … |
| Sex | |  |  |  |  |  |  |  |  | |  |  | |  |  |
|  | Male | 57 | 57.0 | 148 | 49.3 | 1.4 | 0.86, 2.2 | 36 | 52.9 | | 97 | 44.1 | | 1.4 | 0.82, 2.5 |
|  | Female | 43 | 43.0 | 152 | 50.6 | Ref |  | 32 | 47.1 | | 123 | 55.9 | | Ref | … |
| Antibiotics given 6 months prior | |  |  |  |  |  |  |  |  | |  |  | |  |  |
|  | Yes | 52 | 52.0 | 23 | 7.7 | 13.0 | 7.3, 23.3 | 39 | 57.4 | | 18 | 8.2 | | 15.1 | 7.6, 29.8 |
|  | No | 48 | 48.0 | 277 | 92.3 | Ref | … | 29 | 42.7 | | 202 | 91.8 | | Ref | … |
| Comorbidities | |  |  |  |  |  |  |  |  | |  |  | |  |  |
|  | Severity level 1 | 23 | 23.0 | 41 | 13.7 | 2.9 | 1.6, 5.3 | 11 | 16.2 | | 29 | 13.2 | | 2.0 | 0.92, 4.5 |
|  | Severity level 2 | 9 | 9.0 | 22 | 7.3 | 2.1 | 0.91, 4.9 | 7 | 10.3 | | 14 | 6.4 | | 2.7 | 1.0, 7.2 |
|  | Severity level 3 | 25 | 25.0 | 14 | 4.7 | 9.3 | 4.5, 19.2 | 19 | 27.9 | | 10 | 4.6 | | 10.2 | 4.3, 24.1 |
|  | None | 43 | 43.0 | 223 | 74.3 | Ref | … | 31 | 45.6 | | 167 | 75.9 | | Ref | … |
| Surgery | |  |  |  |  |  |  |  | | |  | | |  |  |
|  | Colorectal | 8 | 8.0 | 32 | 10.7 | 0.73 | 0.31, 1.7 | 8 | | 11.8 | 24 | | 10.9 | 0.96 | 0.38, 2.4 |
|  | Orthopedic | 22 | 22.0 | 50 | 16.7 | 1.3 | 0.68, 2.4 | 15 | | 22.1 | 43 | | 19.6 | 1.0 | 0.48, 2.1 |
|  | Vascular, cardiac, thoracic | 30 | 30.0 | 88 | 29.3 | 0.99 | 0.57, 1.7 | 16 | | 23.5 | 56 | | 25.5 | 0.82 | 0.40, 1.7 |
|  | Neurosurgery | 4 | 4.0 | 10 | 3.3 | 1.2 | 0.34, 4.0 | 4 | | 5.9 | 10 | | 4.6 | 1.2 | 0.33, 4.0 |
|  | Hysterectomy | 0 | 0.0 | 15 | 5.0 | Und | … | 0 | | 0.0 | 15 | | 6.8 | Und | … |
|  | Abdominal, pelvic | 36 | 36.0 | 105 | 35.0 | Ref | … | 25 | | 36.8 | 72 | | 32.7 | Ref | … |
| **Risk Factor (Discreet)** | |  | |  | |  | |  | | |  | | | **OR** | **95%CI** |
| Age (years) | |  | |  | | 1.0 | 0.99, 1.0 |  | | |  | | | 1.0 | 0.99, 1.0 |
| Length of Stay (days) | |  | |  | | 1.1 | 1.0, 1.1 |  | | |  | | | 1.0 | 1.0, 1.1 |
