# Supplementary material for: Antibiotic prophylaxis for surgical site infections as a risk factor for infection with Clostridium difficile
Source: PLoS One. 2017 Jun 16;12(6):e0179117. doi: 10.1371/journal.pone.0179117 (PMC5473553; doi:10.1371/journal.pone.0179117)
Supplement: S4 Table — (DOCX) [file pone.0179117.s004.docx]

**S4 Table. Adjusted odds ratios (OR) and 95% confidence intervals (CI) for risk factors independently associated with case status.**

|  | | **Full Sample** | | | **Subset Surgery upon Admission** | | |
| --- | --- | --- | --- | --- | --- | --- | --- |
| **Parameter** | | **P-value** | **OR** | **95% CI** | **P-value** | **OR** | **95% CI** |
| Recommended Antibiotic Use | | <0.01 | 7.6 | 3.9, 14.7 | <0.01 | 6.7 | 2.9, 15.5 |
| Comorbidity Severity | |  |  |  |  |  |  |
|  | Level 1 | 0.15 | 1.8 | 0.81, 3.9 | 0.82 | 0.88 | 0.30, 2.6 |
|  | Level 2 | 0.03 | 3.3 | 1.1, 9.6 | 0.04 | 3.8 | 1.1, 13.9 |
|  | Level 3 | <0.01 | 5.7 | 2.3, 14.2 | 0.01 | 4.0 | 1.4, 11.7 |
| Prior Antibiotics | | <0.01 | 16.1 | 7.7, 34.0 | <0.01 | 19.1 | 8.0, 45.7 |
| Surgery Type | |  |  |  |  |  |  |
|  | Colorectal | 0.97 | 0.98 | 0.34, 2.8 | 0.55 | 1.4 | 0.4, 4.7 |
|  | Orthopedic | 0.01 | 3.4 | 1.4, 8.1 | 0.03 | 3.4 | 1.1, 10.0 |
|  | Vascular, cardiac, thoracic | 0.31 | 1.5 | 0.70, 3.1 | 0.81 | 1.1 | 0.44, 2.9 |
|  | Neurosurgery | 0.66 | 1.5 | 0.27, 7.8 | 0.87 | 1.2 | 0.2, 6.4 |
|  | Abdominal, pelvic | … | Ref | … | … | Ref | … |
