# Supplementary material for: Antibiotic prophylaxis for surgical site infections as a risk factor for infection with Clostridium difficile
Source: PLoS One. 2017 Jun 16;12(6):e0179117. doi: 10.1371/journal.pone.0179117 (PMC5473553; doi:10.1371/journal.pone.0179117)
Supplement: S5 Table — (DOCX) [file pone.0179117.s005.docx]

**S5 Table.** List of eligible surgeries as defined by ICD-9 procedure code.

| **ICD-9 Code** | **Surgical Procedure** |
| --- | --- |
| 00.50 | Implantation of cardiac resynchronization pacemaker without mention of defibrillation, total system |
| 00.51 | Implantation of cardiac resynchronization defibrillator, total system |
| 00.61 | Percutaneous angioplasty or atherectomy of precerebral (extracranial) vessel(s) |
| 00.62 | Percutaneous angioplasty or atherectomy of intracranial vessel(s) |
| 00.63 | Percutaneous insertion of carotid artery stent(s) |
| 00.66 | Percutaneous transluminal coronary angioplasty [PTCA] or coronary atherectomy |
| 00.81 | Revision of knee replacement, tibial component |
| 2.12 | Other repair of cerebral meninges |
| 2.2 | Ventriculostomy |
| 3.4 | Excision or destruction of lesion of spinal cord or spinal meninges |
| 03.53 | Repair of vertebral fracture |
| 07.22 | Unilateral adrenalectomy |
| 17.31 | Laparoscopic multiple segmental resection of large intestine |
| 17.32 | Laparoscopic cecectomy |
| 17.33 | Laparoscopic right hemicolectomy |
| 17.35 | Laparoscopic left hemicolectomy |
| 17.36 | Laparoscopic sigmoidectomy |
| 17.39 | Other laparoscopic partial excision of large intestine |
| 17.55 | Transluminal coronary atherectomy |
| 17.56 | Atherectomy of other non-coronary vessels |
| 31.1 | Temporary tracheostomy |
| 33.27 | Closed endoscopic biopsy of lung |
| 33.52 | Bilateral lung transplantation |
| 34.01 | Incision of chest wall |
| 34.02 | Exploratory thoracotomy |
| 34.2 | Diagnostic procedures on chest wall, pleura, mediastinum, and diaphragm |
| 34.51 | Decortication of lung |
| 34.91 | Thoracentesis |
| 35 | Closed heart valvotomy |
| 35.12 | Open heart valvuloplasty of mitral valve without replacement |
| 35.21 | Replacement of aortic valve with tissue graft |
| 35.22 | Other replacement of aortic valve |
| 35.23 | Replacement of mitral valve with tissue graft |
| 35.24 | Other replacement of mitral valve |
| 35.52 | Repair of atrial septal defect with prosthesis, closed technique |
| 35.53 | Repair of ventricular septal defect with prosthesis, open technique |
| 35.71 | Other and unspecified repair of atrial septal defect |
| 35.72 | Other and unspecified repair of ventricular septal defect |
| 35.96 | Percutaneous valvuloplasty |
| 36.07 | Insertion of drug-eluting coronary artery stent(s) |
| 36.09 | Other removal of coronary artery obstruction |
| 36.11 | (Aorto)coronary bypass of one coronary artery |
| 36.12 | (Aorto)coronary bypass of two coronary arteries |
| 36.12 |  |
| 36.13 | (Aorto)coronary bypass of three coronary arteries |
| 36.14 | (Aorto)coronary bypass of four or more coronary arteries |
| 36.15 | Single internal mammary-coronary artery bypass |
| 36.16 | Double internal mammary-coronary artery bypass |
| 37.25 | Biopsy of heart |
| 37.33 | Excision or destruction of other lesion or tissue of heart,open approach |
| 37.34 | Excision or destruction of other lesion or tissue of heart,other approach |
| 37.51 | Heart transplantation |
| 37.61 | Implant of pulsation balloon |
| 37.63 | Repair of heart assist system |
| 37.66 | Insertion of implantable heart assist system |
| 37.68 | Insertion of percutaneous external heart assist device |
| 37.71 | Initial insertion of transvenous lead [electrode] into ventricle |
| 37.72 | Initial insertion of transvenous leads [electrodes] into atrium and ventricle |
| 37.79 | Revision or relocation of cardiac device pocket |
| 37.80 | Insertion of permanent pacemaker, initial or replacement, type of device not specified |
| 37.81 | Initial insertion of single-chamber device, not specified as rate responsive |
| 37.82 | Initial insertion of single-chamber device, rate responsive |
| 37.83 | Initial insertion of dual-chamber device |
| 37.85 | Replacement of any type pacemaker device with single-chamber device, not specified as rate responsive |
| 37.86 | Replacement of any type of pacemaker device with single-chamber device, rate responsive |
| 37.87 | Replacement of any type pacemaker device with dual-chamber device |
| 37.89 | Revision or removal of pacemaker device |
| 37.94 | Implantation or replacement of automatic cardioverter/defibrillator, total system [AICD] |
| 38 | Incision of vessel |
| 38.11 | Endarterectomy |
| 38.7 | Interruption of the vena cava |
| 38.81 | Other surgical occlusion of vessels |
| 38.91 | Puncture of vessel |
| 39.1 | Intra-abdominal venous shunt |
| 39.25 | Aorta-iliac-femoral bypass |
| 39.27 | Arteriovenostomy for renal dialysis |
| 39.29 | Other (peripheral) vascular shunt or bypass |
| 39.31 | Suture of artery |
| 39.50 | Angioplasty or atherectomy of other non-coronary vessel(s) |
| 39.52 | Angioplasty or atherectomy of other non-coronary vessel(s) |
| 39.53 | Repair of arteriovenous fistula |
| 39.65 | Extracorporeal membrane oxygenation [ECMO] |
| 39.71 | Endovascular implantation of graft in abdominal aorta |
| 39.74 | Endovascular removal of obstruction from head and neck vessel(s) |
| 39.79 | Other endovascular repair (of aneurysm) of other vessels |
| 39.93 | Insertion of vessel-to-vessel cannula |
| 39.95 | Hemodialysis |
| 40.9 | Other operations on lymphatic structures |
| 41.5 | Total splenectomy |
| 43.7 | Partial gastrectomy with anastomosis to jejunum |
| 43.8 | Other partial gastrectomy |
| 44.22 | Endoscopic dilation of pylorus |
| 44.38 | Laparoscopic gastroenterostomy |
| 44.39 | Other gastroenterostomy |
| 44.42 | Suture of duodenal ulcer site |
| 44.43 | Endoscopic control of gastric or duodenal bleeding |
| 44.66 | Other procedures for creation of esophagogastric sphincteric competence |
| 44.67 | Laparoscopic procedures for creation of esophagogastric sphincteric competence |
| 45.02 | Other incision of small intestine |
| 45.13 | Other endoscopy of small intestine |
| 45.16 | Esophagogastroduodenoscopy [EGD] with closed biopsy |
| 45.25 | Closed [endoscopic] biopsy of large intestine |
| 45.31 | Other local excision of lesion of duodenum |
| 45.61 | Multiple segmental resection of small intestine |
| 45.62 | Other partial resection of small intestine |
| 45.71 | Multiple segmental resection of large intestine |
| 45.72 | Cecectomy |
| 45.73 | Right hemicolectomy |
| 45.74 | Resection of transverse colon |
| 45.75 | Left hemicolectomy |
| 45.76 | Sigmoidectomy |
| 45.79 | Other partial excision of large intestine |
| 45.8 | Total intra-abdominal colectomy |
| 45.93 | Other small-to-large intestinal anastomosis |
| 45.94 | Large-to-large intestinal anastomosis |
| 45.95 | Anastomosis to anus |
| 46.01 | Exteriorization of small intestine |
| 46.02 | Resection of exteriorized segment of small intestine |
| 46.10 | Colostomy, not otherwise specified |
| 46.20 | Ileostomy, not otherwise specified |
| 46.42 | Repair of pericolostomy hernia |
| 46.43 | Other revision of stoma of large intestine |
| 46.51 | Closure of stoma of small intestine |
| 46.52 | Closure of stoma of large intestine |
| 47.01 | Laparoscopic appendectomy |
| 47.09 | Other appendectomy |
| 48.63 | Other anterior resection of rectum |
| 48.69 | Other partial proctectomy |
| 48.71 | Suture of laceration of rectum |
| 48.75 | Abdominal proctopexy |
| 48.76 | Other proctopexy |
| 50.11 | Closed (percutaneous) [needle] biopsy of liver |
| 50.12 | Open biopsy of liver |
| 50.21 | Marsupialization of lesion of liver |
| 50.22 | Partial hepatectomy |
| 50.25 | Laparoscopic ablation of liver lesion or tissue |
| 50.29 | Other destruction of lesion of liver |
| 50.3 | Lobectomy of liver |
| 50.59 | Other transplant of liver |
| 51.10 | Endoscopic retrograde cholangiopancreatography [ERCP] |
| 51.22 | Cholecystectomy |
| 51.23 | Laparoscopic cholecystectomy |
| 51.36 | Choledochoenterostomy |
| 51.37 | Anastomosis of hepatic duct to gastrointestinal tract |
| 51.41 | Common duct exploration for removal of calculus |
| 51.43 | Insertion of choledochohepatic tube for decompression |
| 51.49 | Incision of other bile ducts for relief of obstruction |
| 51.59 | Incision of other bile duct |
| 51.61 | Excision of cystic duct remnant |
| 51.69 | Excision of other bile duct |
| 51.79 | Repair of other bile ducts |
| 51.82 | Pancreatic sphincterotomy |
| 51.84 | Endoscopic dilation of ampulla and biliary duct |
| 51.85 | Endoscopic sphincterotomy and papillotomy |
| 51.87 | Endoscopic insertion of stent (tube) into bile duct |
| 51.88 | Endoscopic removal of stone(s) from biliary tract |
| 52.01 | Drainage of pancreatic cyst by catheter |
| 52.09 | Other pancreatotomy |
| 52.13 | Endoscopic retrograde pancreatography [ERP] |
| 52.22 | Other excision or destruction of lesion or tissue of pancreas or pancreatic duct |
| 52.4 | Internal drainage of pancreatic cyst |
| 52.52 | Distal pancreatectomy |
| 52.53 | Radical subtotal pancreatectomy |
| 52.7 | Radical pancreaticoduodenectomy |
| 52.93 | Endoscopic insertion of stent (tube) into pancreatic duct |
| 52.94 | Endoscopic removal of stone(s) from pancreatic duct |
| 53.03 | Repair of direct inguinal hernia with graft or prosthesis |
| 53.05 | Repair of inguinal hernia with graft or prosthesis, not otherwise specified |
| 53.41 | Repair of umbilical hernia with prosthesis |
| 53.4 | Repair of umbilical hernia |
| 53.61 | Incisional hernia repair with prosthesis |
| 53.6 | Repair of other hernia of anterior abdominal wall with graft or prosthesis |
| 53.7 | Repair of diaphragmatic hernia, abdominal approach |
| 54 | Incision of abdominal wall |
| 54.12 | Reopening of recent laparotomy site |
| 54.21 | Laparoscopy |
| 54.23 | Biopsy of peritoneum |
| 54.25 | Peritoneal lavage |
| 54.3 | Excision or destruction of lesion or tissue of abdominal wall or umbilicus |
| 54.4 | Excision or destruction of peritoneal tissue |
| 54.51 | Laparoscopic lysis of peritoneal adhesions |
| 54.59 | Other lysis of peritoneal adhesions |
| 54.91 | Percutaneous abdominal drainage |
| 54.92 | Removal of foreign body from peritoneal cavity |
| 55.03 | Percutaneous nephrostomy without fragmentation |
| 55.4 | Partial nephrectomy |
| 55.51 | Nephroureterectomy |
| 55.53 | Removal of transplanted or rejected kidney |
| 55.69 | Other kidney transplantation |
| 56.0 | Transurethral removal of obstruction from ureter and renal pelvis |
| 57.71 | Radical cystectomy |
| 57.82 | Closure of cystostomy |
| 57.83 | Repair of fistula involving bladder and intestine |
| 60.69 | Other prostatectomy |
| 65.63 | Laparoscopic removal of both ovaries and tubes at same operative episode |
| 68.31 | Laparoscopic supracervical hysterectomy [LSH] |
| 68.39 | Other and unspecified subtotal abdominal hysterectomy |
| 68.41 | Total laparoscopic hysterectomy [TLH] |
| 68.49 | Hysterectomy |
| 68.51 | Laparoscopically assisted vaginal hysterectomy (LAVH) |
| 68.59 | Other and unspecified vaginal hysterectomy |
| 68.71 | Radical vaginal hysterectomy |
| 68.79 | Hysterocolpectomy |
| 69.02 | Dilation and curettage following delivery or abortion |
| 70.50 | Repair of cystocele and rectocele |
| 70.72 | Repair of colovaginal fistula |
| 74.1 | Lower uterine segment cesarean section |
| 77.4 | biopsy of bone |
| 78.5 | Internal fixation of bone without fracture reduction |
| 78.6 | Removal of implanted devices from bone |
| 79.1 | Closed reduction of fracture with internal fixation |
| 79.3 | Open reduction of fracture with internal fixation |
| 80.1 | Other arthrotomy |
| 80.3 | Biopsy of joint structure |
| 80.51 | Excision of intervertebral disc |
| 81.00 | Spinal fusion, not otherwise specified |
| 81.01 | Atlas-axis spinal fusion |
| 81.02 | Other cervical fusion, anterior technique |
| 81.03 | Other cervical fusion, posterior technique |
| 81.04 | Dorsal and dorsolumbar fusion, anterior technique |
| 81.05 | Dorsal and dorsolumbar fusion, posterior technique |
| 81.06 | Lumbar and lumbosacral fusion, anterior technique |
| 81.07 | Lumbar and lumbosacral fusion, lateral transverse process technique |
| 81.08 | Lumbar and lumbosacral fusion, posterior technique |
| 81.32 | Refusion of other cervical spine, anterior technique |
| 81.35 | Refusion of dorsal and dorsolumbar spine, posterior technique |
| 81.36 | Refusion of lumbar and lumbosacral spine, anterior technique |
| 81.37 | Refusion of lumbar and lumbosacral spine, lateral transverse process technique |
| 81.38 | Refusion of lumbar and lumbosacral spine, posterior technique |
| 81.51 | Total hip replacement |
| 81.52 | Partial hip replacement |
| 81.53 | Revision of hip replacement, not otherwise specified |
| 81.54 | Total knee replacement |
| 81.55 | Revision of knee replacement,not otherwise specified |
| 81.65 | Vertebroplasty |
| 81.66 | Kyphoplasty |
| 83.39 | Excision of lesion of other soft tissue |
| 83.88 | Other plastic operations on tendon |
| 84.11 | Amputation of toe |
| 84.12 | Amputation through foot |
| 84.15 | Other amputation below knee |
| 84.17 | Amputation above knee |
| 84.3 | Revision of amputation stump |
| 85.41 | Unilateral simple mastectomy |
| 86.01 | Aspiration of skin and subcutaneous tissue |
| 86.05 | Incision with removal of foreign body or device from skin and subcutaneous tissue |
| 86.07 | Insertion of totally implantable vascular access device [VAD] |
| 86.22 | Excisional debridement of wound, infection, or burn |
| 86.69 | Other skin graft to other sites |
| 87.51 | Percutaneous hepatic cholangiogram |
| 87.53 | Intraoperative cholangiogram |
| 87.54 | Other cholangiogram |
| 96.04 | Insertion of endotracheal tube |
| 97.55 | Removal of T-tube, other bile duct tube, or liver tube |
